# Existing Datasets to Study the Impact of Internal Migration on Caregiving Arrangements

**DOI:** 10.1093/geroni/igaa057.2703

**Published:** 2020-12-16

**Authors:** Hanzhang Xu, Yaolin Pei, Matthew Dupre, Bei Wu

**Affiliations:** 1 Duke University School of Medicine, Durham, North Carolina, United States; 2 New York University, New York, New York, United States; 3 Duke University, Durham, North Carolina, United States

## Abstract

Massive rural-to-urban migration in China has a significant impact on caregiving arrangements among Chinese older adults. To stimulate research on the intersection of migration and caregiving, we conducted an inventory of longitudinal aging survey datasets that included older adults from mainland China. Large public available datasets that included measures related to migration and caregiving were searched and reviewed for eligibility. Key characteristics of each dataset, including study design, sample size, and measures, were extracted. Seven eligible datasets were identified, and five included national representative samples. Measures for migration varied across datasets. Some datasets included information on the migration history of older adults, whereas others focused on the migration of adult children. Similarly, caregiving was measured using different questions in each dataset. Caregiving activities were assessed with regard to their type, source, and amount. High-quality datasets exist to support research on migration and caregiving arrangements among Chinese older adults.

